# Associations between metabolic and cardiovascular biomarkers and cognition in a nationally representative sample

**DOI:** 10.1093/geroni/igaf122.3227

**Published:** 2025-12-31

**Authors:** Adea Rich, Sheina Emrani, Kristen George, Jennifer Manly, Richard Jones, Zachary Kunicki

**Affiliations:** Columbia University, New York, New York, United States; University of Pennsylvania, Philadelphia, Pennsylvania, United States; University of California, Davis, Davis, California, United States; Columbia University Irving Medical Center, New York, New York, United States; Brown University, Brown University, Rhode Island, United States; Warren Alpert Medical of Brown University, Providence, Rhode Island, United States

## Abstract

Inflammatory, metabolic, and cardiovascular dysregulation contribute to cognitive impairment and Alzheimer’s disease and Alzheimer’s disease and related dementias (AD/ADRD) in older adults. AD/ADRD blood-based biomarkers are a cost-effective and accessible tool for diagnosis and monitoring, and routine blood biomarkers may offer additional insights. However, their associations with high-quality cognitive assessments remain understudied in nationally representative samples. We analyzed 1,335 older adults (mean age: 74.2; 57% female) from the 2016 Health and Retirement Study (HRS) biomarker data and Harmonized Cognitive Assessment Protocol (HCAP). Biomarkers included total cholesterol, high-density lipoprotein, hemoglobin A1C, c-reactive protein, and cystatin C. Cognitive domains (memory, executive function, language fluency) and diagnostic classifications (normal, mild cognitive impairment [MCI], AD/ADRD) were determined using the Manly-Jones algorithm. Missing data were handled with multiple imputation, and survey weights were incorporated in all analyses. We assessed biomarker levels across diagnoses. Linear regression models examined associations between biomarkers and cognitive domains, and ordinal logistic regression assessed associations between biomarkers and HCAP diagnosis. Models adjusted for age, sex, education, and race/ethnicity. Cystatin C differed across diagnostic groups (p = 0.004), being higher in MCI than normal (p < 0.01). Higher cystatin C was linked to poorer language fluency (β = -0.08, p = 0.02) and executive functioning (β = -0.07, p = 0.02). Higher A1C was also associated with lower executive functioning (β = -0.07, p = 0.02). Elevated cystatin C and A1C can indicate inflammation and poor cardiovascular and metabolic health, which may contribute to impairments in executive function and language fluency domains.

